# Streaming Feature Selection for Multi-Label Data with Dynamic Sliding Windows and Feature Repulsion Loss

**DOI:** 10.3390/e21121151

**Published:** 2019-11-25

**Authors:** Yu Li, Yusheng Cheng

**Affiliations:** 1 School of Computer and Information, Anqing Normal University, Anqing 246003, China; liyu_92@163.com; 2 Lab of Multimedia and Recommendation Systems, Hefei University of Technology, Hefei 230009, China; 3 The University Key Laboratory of Intelligent Perception and Computing of Anhui Province, Anqing 246003, China

**Keywords:** multi-label learning, streaming feature selection, sliding window, feature repulsion loss

## Abstract

In recent years, there has been a growing interest in the problem of multi-label streaming feature selection with no prior knowledge of the feature space. However, the algorithms proposed to handle this problem seldom consider the group structure of streaming features. Another shortcoming arises from the fact that few studies have addressed atomic feature models, and particularly, few have measured the attraction and repulsion between features. To remedy these shortcomings, we develop the streaming feature selection algorithm with dynamic sliding windows and feature repulsion loss (SF-DSW-FRL). This algorithm is essentially carried out in three consecutive steps. Firstly, within dynamic sliding windows, candidate streaming features that are strongly related to the labels in different feature groups are selected and stored in a fixed sliding window. Then, the interaction between features is measured by a loss function inspired by the mutual repulsion and attraction between atoms in physics. Specifically, one feature attraction term and two feature repulsion terms are constructed and combined to create the feature repulsion loss function. Finally, for the fixed sliding window, the best feature subset is selected according to this loss function. The effectiveness of the proposed algorithm is demonstrated through experiments on several multi-label datasets, statistical hypothesis testing, and stability analysis.

## 1. Introduction

In recent years, multi-label learning has been extensively used in various practical applications, such as text classification [1] and gene function classification [2]. While a sample has only one label in the traditional single-label learning, a sample can be associated with multiple labels simultaneously in a multi-label learning framework. However, high-dimensional features of multi-label datasets inevitably include uncorrelated or redundant features, which result in low classification performance and overfitting. To alleviate this problem, a number of methods for multi-label learning with dimensionality reduction have been proposed. Based on whether the reduction is achieved through changes to the feature extraction or selection stages, these methods can be generally divided into two categories, namely multi-label feature extraction methods [3] and multi-label feature selection methods. Multi-label feature extraction is a method that converts original high-dimensional feature space into a new low-dimensional feature space through transforming or mapping, and the new constructed features are usually combinations of original features. Multi-label feature selection selects the feature subset from the original feature space directly, and keeps the physical meaning for the selected features. In this work, we focus mainly on the latter. 

Feature selection effectively addresses the curse of dimensionality [4,5,6] by removing redundant or uncorrelated features, reducing the feature space dimensionality, and keeping a feature subset that contains all or most of the information in the original feature space. Thereby, feature selection shortens classification times and typically improves classification accuracies. Multi-label feature selection methods are usually classified into three main groups: filtering methods [7,8], wrapper methods [9], and embedded methods [10]. While filtering methods perform classifier-independent feature selection, wrapper methods use the accuracy of a given classifier to determine the quality of the selected features. Embedded methods achieve model fitting and feature selection simultaneously. In this paper, we consider only filtering methods which seek to design effective metrics for evaluating the importance of candidate features. Such metrics can be based on information entropy [11,12,13,14], the classification boundary [15], and rough sets [16,17]. In particular, Lee et al. [18] proposed a multivariate mutual information criterion for multi-label feature selection. Lee and Kim [19] approached fast multi-label feature selection using an information-theoretic feature ranking, which employs a score function to assess the importance of each feature. Lin et al. [20] proposed a multi-label feature selection algorithm based on the neighborhood mutual information. In this algorithm, an effective feature subset is selected according to the maximum correlation and minimum redundancy criteria. This method generalizes the neighborhood entropy function of single-label learning to multi-label learning. 

Nevertheless, the aforementioned algorithms assume full knowledge of the entire feature space, while features emerge incrementally or dynamically in numerous modern applications [11,21,22]. For example, hot news is constantly updated with different keywords in each piece of news, indicating that features of every data sample cannot be necessarily available in advance. In such cases, the full feature set is not a priori known at the beginning of the learning phase, and the features are referred to as streaming (or online) features. Traditional feature selection methods have been designed for offline features which are fully known in advance, however, not for streaming features. Hence, the problem of how to perform feature selection in dynamic environments has witnessed growing attention. On the one hand, sliding-window mechanisms represent one key module for dealing with this problem. Indeed, most of the streaming-data algorithms [23,24] are based on fixed-size windows. While such windows are simple, they cannot be realistically applied because of the variations in the streaming feature generation and velocity. On the other hand, most of the existing methods for multi-label feature selection evaluate features separately and overlook the feature group structure and interactions. 

The group structure of streaming features is the set of all features generated at each time instance. This structure can be regarded as prior knowledge, which may improve the learning performance of streaming-data algorithms. While the group structure has been exploited for feature selection in single-label learning [25,26], few approaches have been proposed for incorporating this structural information in streaming feature selection algorithms for multi-label learning [21,27]. Due to the temporal variability of the group structure size, we use a dynamic sliding window for group feature processing. In particular, we select the features that have a strong correlation with the data labels and store these features in a fixed sliding window. When the fixed window reaches a certain threshold, we re-evaluate the correlation between all candidate and selected features. Essentially, the streaming feature selection [28] problem is handled in this paper through a combination of fixed and dynamic sliding windows. 

Indeed, while recent algorithms for multi-label streaming feature selection can reasonably select a subset of features for multi-label classification learning, these algorithms don’t exploit the feature interactions. For example, Lin et al. [29] proposed a fuzzy mutual information criterion for streaming feature selection and multi-label learning. Also, Almusallam et al. [30] introduced an efficient algorithm for unsupervised feature selection for streaming features. However, both methods fail to consider the relationships and interactions of features in the feature selection process [31,32]. Feature interactions can be interpreted as follows. On the one hand, if the performance of a classification algorithm can be improved by the combination of two features, then we can say that there is a positive correlation force between the features. On the other hand, if the combination of the two features reduces the classification performance, then there is a negative correlation force between the features. If such features are regarded as atoms, this interaction force becomes similar to the mutual attraction between magnetic poles in physics. One relevant work is that of Reyes et al. [33] who proposed an effective lazy learning algorithm based on a data gravitation model for multi-label learning. In this algorithm, each data instance is regarded as an atom within a data gravitation model, which led to sound results in multi-label learning. However, this algorithm considers only the attraction, and not the repulsion between samples. In this paper, we propose the inclusion of a novel feature repulsion loss term in the data gravitation model. More specifically, this paper proposes a multi-label streaming feature selection algorithm based on dynamic sliding windows and feature repulsion loss. 

Firstly, the variable-size and fixed-size sliding windows are respectively set as the processing and cache windows. The size of the dynamic sliding window is automatically adjusted according to the number of features generated in each time interval. The streaming features are examined in the processing window, and thus the satisfactory candidate features are temporarily saved to the cache window. Based on the neighborhood information entropy, feature attraction and repulsion terms are defined and combined together to construct the feature repulsion loss function. When the cache window reaches a certain threshold, the repulsion loss is evaluated for all candidate features in the cache window, and those features with the largest feature repulsion loss (FRL) are saved to the selected feature subset. The window slides backward sequentially until all features are in the sliding window. Finally, through extensive experimental comparison between the proposed method and other state-of-the-art methods, we show that the proposed algorithm is more effective. This conclusion is supported by statistical hypothesis testing and stability analysis.

The rest of the paper is organized as follows. Related work is surveyed in Section 2. Section 3 briefly reviews multi-label learning, the neighborhood information entropy, and the neighborhood conditional mutual information. Section 4 introduces the sliding window scheme, the feature repulsion loss, and hence the details of our proposed streaming feature selection algorithm with dynamic sliding windows and feature repulsion loss (SF-DSW-FRL) method for multi-label feature selection. In Section 5, experimental results on publically available multi-label datasets show that our SF-DSW-FRL algorithm is more effective than other competing algorithms. Statistical hypothesis tests further verify the validity of our method in Section 5. In the last section, we conclude our discussion and point out further research directions.

## 2. Related Work

Data samples in multi-label learning problems are usually described with high-dimensional features. Offline and online approaches may be adopted for multi-label feature selection. For offline approaches, all features are assumed to be available in advance before the initiation of feature selection. However, in real-world scenarios, features are actually generated dynamically in a streaming manner. Online approaches handle this situation where there is no prior knowledge of all features at the onset of the learning phase.

In practical applications, numerous types of data are generated in real time [34,35]. Such data types include log records or click streams in web, email, and blog applications. Within the framework of multi-label learning, many algorithms have been proposed to handle streaming data. For example, Read et al. [36] proposed a scalable and efficient multi-label classification approach for evolving data streams, where multi-label pruned-set classifiers are applied at the leaves of Hoeffding trees. This approach inherits the high performance of incremental decision trees, and the predictive power of efficient multi-label methods. An adaptive windowing (ADWIN) bagging method is used to allow adaption to concept drift in evolving data. This approach makes real-time predictions and updates the learned model for every example. Eskandari et al. [11] proposed an online feature selection method based on rough sets. Liu et al. [21] proposed an online multi-label group feature selection. This method consists of two-phase: online group selection and online inter-group selection. In the group selection, feature groups which is important to label set are selected according to a criterion design by themselves. In the inter-group selection, feature subset is selected by considering feature interaction and feature redundancy.

Not only does multi-label feature selection face the problem of handling streaming features, but also faces label-space challenges, such as missing, weak, and unbalanced labels. Lin et al. [37] proposed a multi-label feature selection method for streaming labels, i.e., the labels arrive one at a time. In this case, the learning task is to arrange features iteratively and select the best feature subset as new labels arrive. While feature selection in dynamic environments has witnessed growing attention, higher performance efficient algorithms are still needed. Our work is one step in this direction. 

## 3. Neighborhood Information Entropy for Multi-Label Learning

### 3.1. Multi-Label Learning

In a multi-label learning framework, let $X=R^{q\times m}$ represent the feature space, where the sample set is $X=\left\{x_{1},x_{2},\ldots ,x_{q}\right\}$, the label space is $L=\left\{l_{1},l_{2},\ldots ,l_{n}\right\}$, and the sample $x_{i}$ can be described with an *m*-dimensional feature vector, $x_{i}=\left[x_{i1},x_{i2},\ldots ,x_{im}\right]$. Let the label of the sample $x_{i}$ in the label space be denoted as $Y_{i}=\left[y_{i1},y_{i2},\ldots ,y_{in}\right]$, where $y_{ia}=1$ if $x_{i}$ has a class label $l_{a}$ and $y_{ia}=0$, otherwise.

In Table 1, the sample $x_{1}$ has the multiple labels $l_{1}$ and $l_{3}$. Equivalently, we write 1 under these labels for $x_{1}$ in the binary representation of the multiple labels (See Table 2). By contrast, we write 0 under the label $l_{2}$ for $x_{1}$, which means that the sample $x_{1}$ does not have this label. For the samples $x_{2}$, $x_{3}$ and $x_{4}$, entries in Table 2 are made with the same reasoning.

### 3.2. Neighborhood Information Entropy

Given a set of objects $X=\left\{x_{1},x_{2},\ldots ,x_{q}\right\}$, we define a symbol $\Delta \left(x_{i},x_{j}\right)$, representing the distance between $x_{i}$ and $x_{j}$. When δ≥0 is given, $\delta \left(x_{i}\right)=\left\{x_{j}|\Delta \left(x_{i},x_{j}\right)\leq \delta \right\}$ represents all neighborhood objects contained in objects $x_{i}$, i.e., the objects with similar feature values should be divided into the same class, and it is clear that $\delta \left(x_{i}\right)$ varies with δ. The neighborhood mutual information is better described in [20].

**Definition** **1.** *Given an object* $x_{i}$ *and a label* $l_{i}\in L$ *, the neighborhood under the label* $l_{i}$ *of the object* $x_{i}$ *can be defined as [20]:*

(1) $$m_{l_{i}}\left(x_{i}\right)=\Delta \left(x_{i},NS_{l_{i}}\left(x_{i}\right)\right)-\Delta \left(x_{i},NT_{l_{i}}\left(x_{i}\right)\right).$$

*where* $NS_{l_{i}}\left(x_{i}\right)$ *represents the nearest miss to the object, and* $NT_{l_{i}}\left(x_{i}\right)$ *represents the nearest hit to the object.*

From Equation (1), we know that the object has a different margin granularity under different labels. Lin [20] transformed the neighborhood entropy function in single-label learning to multi-label learning and proposed the concept of a neutral viewpoint from the cognitive viewpoint.

**Definition** **2.** *Given the object* $x_{i}$ *and the label* $l_{i}$ *, the margin of* $x_{i}$ *based on the neutral viewpoint is formulated as [20]:*

(2) $$m^{neu}\left(x_{i}\right)=\frac{1}{\left|L\right|}\sum_{i=1}^{\left|L\right|}m_{l_{i}}\left(x_{i}\right).$$

**Definition** **3.** *Given a set of objects* $X=\left\{x_{1},x_{2},\ldots x_{q}\right\}$ *,* $x_{i}\in X$ *, the neutral neighborhood with respect to x is defined as [20]:*

(3) $$\delta \left(x_{i}\right)=\left\{x_{j}|\Delta \left(x_{i},x_{j}\right)\leq m^{neu}\left(x_{i}\right)\right\}.$$

**Definition** **4.** *Let*$X=\left\{x_{1},x_{2},\ldots x_{q}\right\}$*and F be the sample and feature sets, respectively. For the features f in F, we denote the neighborhood of*$x_{i}$*by*$\delta_{f}\left(x_{i}\right)$. *The neighborhood information entropy [20] is defined as*

(4) $$NH^{\delta }\left(f\right)=-\frac{1}{q}\sum_{i=1}^{q}log\frac{\left\|\delta_{f}\left(x_{i}\right)\right\|}{q}.$$

*where*$\left\|\delta_{f}\left(x_{i}\right)\right\|$*denotes the number of neighborhood objects of object*$x_{i}$*induced by f.*

**Definition** **5.** *Suppose F is the entire feature space.* *The neighborhood mutual information [20] of the features* $f_{1}\in F$ *and the label space L is defined as*

(5) $$NMI^{\delta }\left(f_{1};L\right)=-\frac{1}{q}\sum_{i=1}^{n}\sum_{j=1}^{q}log\frac{\left\|l_{i}{}_{\left(x_{j}\right)}\right\|}{q}.$$

*where* $\left\|\delta_{f}\left(x_{i}\right)\right\|$ *denotes the number of samples with the same class as sample* $x_{i}$ *by* $f_{1}$ *. According to the definitions in (4), (5), the following formula can be established*

(6) $$NMI^{\delta }\left(f_{1};L\right)=NH^{\delta }\left(f_{1}\right)+NH^{\delta }\left(L\right)-NH^{\delta }\left(f_{1},L\right).$$

Numerous high-performance multi-label feature selection algorithms have been proposed based on the neighborhood mutual information. However, this approach ignores the influence of a single selected feature on candidate features. The neighborhood conditional mutual information overcomes this limitation, and measures the interaction among more than two features. This criterion is defined as follows.

**Definition** **6.** *Suppose F is the entire feature space and S is a feature subset. Assuming that* $f_{1}\in F-S$ *and* $f_{2}\in S$ *, the neighborhood conditional mutual information is defined as*

(7) $$\begin{array}{cc} NMI^{\delta }\left(f_{1};L|f_{2}\right) & =NH^{\delta }\left(f_{1}|f_{2}\right)-NH^{\delta }\left(f_{1}|L,f_{2}\right)\\ & =NH^{\delta }\left(f_{1},f_{2}\right)-NH^{\delta }\left(f_{2}\right)-NH^{\delta }\left(f_{1},f_{2},L\right)+NH^{\delta }\left(f_{2},L\right). \end{array}$$

An extension of the neighborhood conditional mutual information is the minimum neighborhood conditional mutual information, which can effectively measure the redundancy between the pertinent candidate feature and the selected feature subset. This measure is defined as follows.

**Definition** **7.** *Let F be the entire feature space and S be a feature subset. If*$f_{i}\in F-S$*,*$f_{j}\in S$, *the minimum neighborhood conditional mutual information is given by*

(8) $$\min\left(NMI^{\delta }\left(f_{i};L|f_{j}\right)\right).$$

The smaller this measure is, the greater the redundancy between the candidate and selected features is and the weaker the correlation with the label space is. On the contrary, the larger that measure is, the smaller the redundancy between the candidate and selected features is, and the stronger the correlation with the label space is. 

## 4. Streaming Feature Selection with Dynamic Sliding Windows and Feature Repulsion Loss

### 4.1. Sliding-Window Mechanisms

While the data in streaming applications is of a real-time, variable, and large-scale nature, not all of the data is relevant and useful. So, it is necessary to extract these relevant data portions in streaming data environment for further analysis. While traditional feature selection algorithms are not easily applicable in this setting, sliding-window mechanisms represent a viable alternative. These mechanisms generally fall into two types: time-based and tuple-based sliding windows. A time-based sliding window is essentially a dynamic sliding window, for which a collection of ordered elements is arranged by time stamps as a time series, $Q=\left(\left(Q_{1}=\left(F_{1},T_{1}\right)\right),\left(Q_{2}=\left(F_{2},T_{2}\right)\right),\ldots \left(Q_{i}=\left(F_{i},T_{i}\right)\right)\right)$ where $\left(Q_{i}=\left(F_{i},T_{i}\right)\right)$ is the attribute value of the streaming data generated at time *T_i_.*

The window size should be adjusted as follows:

(1) If the number of streaming data samples generated at time *T_i+1_* is greater or less than the window size *W* at time *T_i_*, memory underutilization or insufficient storage space respectively occurs. In this case, the window size should be selected such as $\left|Q_{i+1}\right|\leq W\leq 1.1\times \left|Q_{i+1}\right|$.

(2) If the number of streaming data samples generated at time *T_i+1_* is equal to the window size *W* at time *T_i_*, then the window size is appropriate, and no adjustment is needed.

The tuple-based (or fixed-size) sliding windows sequentially receive the generated features in chronological order. When the number of features in the sliding window reaches the window size, the window is moved forward one position with the entry of a new data feature, while the data at the end of the original window is deleted.

**Figure 1:**
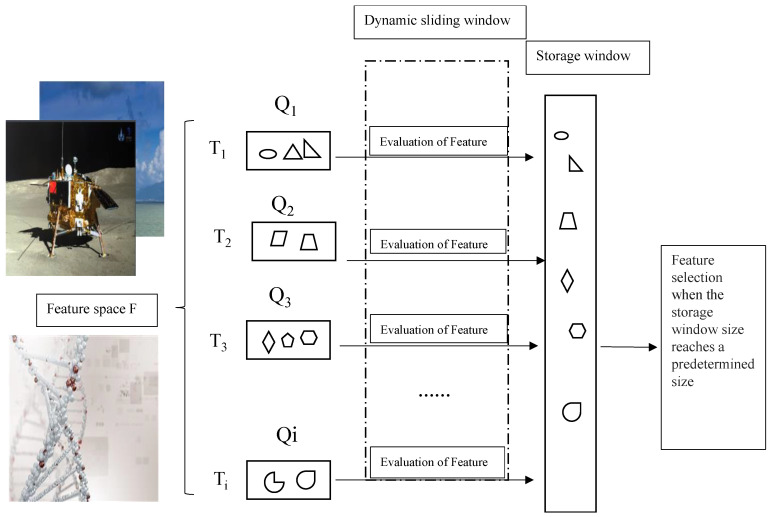
The proposed method for online multi-label feature selection.

Most of the existing streaming-data algorithms use fixed-size sliding windows. If the fixed size of the sliding window is larger than the number of the streaming features generated at the time *T_i_*, the memory is not fully utilized. On the contrary, if the window size is smaller than the number of the streaming features generated at the time *T_i_*, the memory becomes insufficient for streaming feature processing. These limitations can be avoided through using dynamic sliding windows. Moreover, for processing flow features, the proposed method not only considers the feature group structure, but also considers the interaction between the selected and candidate features. Therefore, dynamic and fixed sliding windows are jointly exploited in this paper. The dynamic sliding window is used to pre-process the group streaming features while the important candidates among these features are selected and temporarily stored in the fixed-size window. The size of the dynamic sliding window varies according to the number of feature groups generated at the time *T_i_*. The fixed-size sliding window is used to measure the interaction between the selected and candidate streaming features by computing a feature repulsion loss function and selecting the best feature subset. This process is summarized in Figure 1.

In Figure 1, the different shapes represent different feature types, and the symbols *Q_1_, Q_2_, … , Q_i_* denote groups of features generated at times *T_1_, T_2_, … , and T_i_*, respectively. The generated features at each time instance first enter the dynamic window for pre-processing. The feature that satisfies the pre-processing criteria is temporarily saved to the storage window. When the storage window reaches its size threshold, the optimal feature is saved to the selected feature subset.

### 4.2. Feature Repulsion Loss

By analogy to the mutual repulsion and attraction of magnetic poles, the feature repulsion loss consists of attraction and repulsion terms. Wang et al. [38] firstly proposed this repulsion loss model and successfully applied it to pedestrian detection. For multi-label feature selection, if the feature is considered as an atom, attraction and repulsion forces may exist between the candidate and selected features. Therefore, an in-depth analysis is needed to redefine the feature attraction and repulsion terms and apply the repulsion loss to multi-label feature selection. Mutual information measures are usually adopted for computing the correlation of the candidate features to the label space based on the maximum correlation and minimum redundancy criteria. However, the size of the feature information content is typically ignored. Following the definition of the information gain ratio for the C4.5 decision trees [39], the ratio of the neighborhood mutual information to the self-information is called the feature attraction term (ATC):

(9) $$\mathrm{ATC}=\frac{NMI^{\delta }\left(f_{i};L\right)}{NH^{\delta }\left(f_{i}\right)}.$$

According to Equation (9), a higher attraction of the candidate features to the label space leads to a higher ATC value. While the neighborhood mutual information can effectively measure the correlation between a feature and a label space, the feature attraction term can more reasonably measure the force between a feature and the label space.

As magnetic poles may have mutual repulsion, the candidate features may repel all of the selected features or a single feature. To model this behavior, two feature repulsion terms are proposed. By the minimum conditional mutual information, the redundancy of a candidate feature with respect to the entire selected feature subset is effectively determined. However, in this process, the information of the candidate feature itself is neglected. In multi-label feature selection, the repulsion to features of a subset is defined in order to determine the repulsion between a candidate feature and the entire selected feature subset. The repulsion to a feature subset (RFS) is the ratio of the minimum neighborhood conditional mutual information to self-information:

(10) $$\mathrm{RFS}=\frac{\min_{f_{u}\in S}\left(NMI^{\delta }\left(f_{i};L|f_{u}\right)\right)}{NH^{\delta }\left(f_{i}\right)}.$$

According to Equation (10), larger values of the minimum neighborhood conditional mutual information entropy lead to smaller redundancy between the candidate and selected features, smaller neighborhood self-information entropy, as well as smaller uncertainty of candidate features. Larger RFS values indicate smaller repulsion between a candidate feature and the selected feature subset and hence indicate a higher likelihood of selecting this candidate feature to be in the selected feature subset. In addition, candidate features may also have repulsive effects on a selected feature. To reduce the probability that such candidate features are selected into a feature subset, the repulsive force between features (RFF) is defined as:

(11) $$\mathrm{RFF}={\left(NMI^{\delta }\left(f_{i};L|f_{j}\right)-NMI^{\delta }\left(f_{i};L\right)\right)}^{2}.$$

If the selected feature $f_{j}$ and the candidate feature $f_{i}$ are mutually repulsive, $NMI^{\delta }\left(f_{i};L|f_{j}\right)\leq NMI^{\delta }\left(f_{i};L\right)$, and the *RFF* value becomes smaller. If the selected feature $f_{j}$ and the candidate feature $f_{i}$ attract each other, then $NMI^{\delta }\left(f_{i};L|f_{j}\right)\geq NMI^{\delta }\left(f_{i};L\right)$, and the RFF value becomes larger.

Feature repulsion loss is composed of feature attraction and repulsion terms. In order to select features that attract each other and contain more relevant information, we propose the feature repulsion loss function (*FRL*) that combines feature attraction and feature repulsion.

(12) $${\mathrm{argmax}}_{f_{i}}FRL\left(f_{i},f_{j},f_{u},L\right)=\frac{NMI^{\delta }\left(f_{i};L\right)}{NH^{\delta }\left(f_{i}\right)}\times \frac{\min_{f_{u}\in S}\left(NMI^{\delta }\left(f_{i};L|f_{u}\right)\right)}{NH^{\delta }\left(f_{i}\right)}\times {\left(NMI^{\delta }\left(f_{i};L|f_{j}\right)-NMI^{\delta }\left(f_{i};L\right)\right)}^{2}.$$

According to Equation (12), candidate features corresponding to the maximum FRL should be selected and stored in the feature subset. We know that larger ATC values indicate higher attraction of the candidate features to the label space. A larger RFS value indicates less redundancy between the selected feature subset and the newly selected feature. Also, a larger RFF value reflects a greater interaction between the newly selected feature and the selected feature subset. Combining these two features can effectively improve the classification performance.

### 4.3. Streaming Feature Selection

Next, we present the details of our streaming feature selection algorithm based on a dynamic sliding window and feature repulsion loss. This algorithm assumes that all features are unknown before the feature selection, while the total number of features remains unchanged. The variable-size and fixed-size sliding windows are respectively set as the processing and cache windows. The size of the processing window changes along with the number of streaming features reached at the time interval ΔT. The algorithm is divided into two stages. In the first stage, all of the streaming features generated at time *T_i_* are entered into the processing window. Then, the neighborhood mutual information entropy values of all candidate features in the processing window are computed according to (5). The candidate features whose entropy values are larger than the threshold δ are selected and temporarily saved to the cache window. When the number of features in the cache window reaches the window size, the feature with the maximum neighborhood mutual information in the cache window is selected as the first feature of the feature subset. For the second stage, the generated features are streamed sequentially into the processing window. After the number of features in the cache window reaches the window size again, the value of the feature repulsion loss function is calculated for all candidate features in the cache window according to (12). Hence, the candidate features with the largest feature repulsion loss (FRL) are saved to the selected feature subset. The processing window moves backward sequentially until all the candidate features are in the sliding window. The corresponding pseudo-code is described in Algorithm 1.

**Algorithm 1** Streaming feature selection algorithm with dynamic sliding windows and feature repulsion loss (SF-DSW-FRL)**Input:***F*: feature stream, *L*: a set of labels; *AA*: a fixed window for storing candidate features; *m*: the maximum *AA* capacity; threshold δ **Output: S:** Selected feature subset1) (1)S=∅(2)AA=∅(3)Divide F into *n* parts randomly, denoted by *Q_1_, Q_2_ ,…, Q_n_*;(4)Repeat;(5)Get a group *Q_i_* at time *t_i_*;(6)While |AA|<m(7)\* Select candidate features stored in the storage window*\(8)  for *i* = 1 to *n* do(9)   for *j*=1 to |*Q_i_*| do(10)    if NMI(*Q_ij_*)> δ(11)     *AA*=*AA*∪*Q_ij_*;(12)      if |*AA*|==*m*(13)    \*Selecting features in the storage window*\(14)      for *ii* = 1 to |*AA*| do(15)       Calculate the feature repulsion loss for each feature by Equation (12);(16)      end for(17)     Select the feature *f_ii_* with the largest FRL;(18)     S=S∪{*f_ii_*}(19)     *AA*=*AA*-{*f_ii_*};(20)    end if(21)   end if(22)  end for(23) end for(24)end while;(25)Until no feature groups are available;(26)Return *S*;

### 4.4. Time Complexity

Now, we discuss the time complexity of the proposed algorithm. Let the storage window size be *m*, the label set size be *|L|*, and the feature subset size be |S|. Then, the time complexity of selecting candidate features from the storage window is *O(m|L|)*. When the storage window reaches its threshold, in the while loop, the FRL value of each candidate feature needs to be run *m* times. Totally, the time complexity of the while loop is *O(m|S||L|)*.

## 5. Experimental Setup and Results

### 5.1. The Experimental Datasets

For testing the performance of the SF-DSW-FRL algorithm, we selected eight multi-label datasets from different application areas. These datasets can be downloaded from the Mulan Library (http://mulan.sourceforge.net/datasets.html). Among these, the Arts, Computer, Health, Business, and Education datasets are widely used in web page classification. *Cal500* is a benchmark for music, with 502 samples, 68 features, and 174 labels. The Birds dataset has 645 samples, 260 features, and 20 labels. The Yeast dataset is a biology data set with 2417 samples, 103 features, and 14 labels. Table 3 shows detailed information about the eight multi-label datasets, such as the numbers of samples, features, labels, training samples, and test samples. In our experiments, the full feature space is assumed to be unknown and the features stream in on a group-by-group basis. We use the above datasets of Table 3 to simulate learning scenarios with streaming features and evaluate our proposed method.

### 5.2. Evaluation Criteria

In multi-label classification learning, the performance evaluation for the multi-label learning algorithms is more complicated than that of single-label learning algorithms. For our experimental evaluation, we select the average precision (AP), Hamming loss (HL), one error (OE), ranking loss (RL), and coverage (CV) as evaluation metrics [40]. Given a test dataset $T=\left\{(x_{i},Y_{i})|1\leq i\leq p\right\}$, the set of labels predicted by the proposed algorithm is recorded as h(x). The above evaluation metrics are defined as follows.

#### 5.2.1. Average Precision (AP):

(13) $$\mathrm{AP}\left(f\right)=\frac{1}{p}\sum_{i=1}^{p}\frac{1}{\left|Y_{i}\right|}\sum_{y\in Y_{i}}\frac{\left|\left\{y^{\prime }|rank_{f}\left(x,y^{\prime }\right)\leq rank_{f}\left(x_{i},y\right),y^{\prime }\in Y_{i}\right\}\right|}{rank_{f}\left(x_{i},y\right)}.$$

This evaluation metric gives the average fraction of labels ranked above a particular label y∈Y which actually are in *Y*. The larger the value of the indicator is, the better the performance of the classifier is. The optimal value is 1.

#### 5.2.2. Hamming Loss (HL):

(14) $$\mathrm{HL}\left(h\right)=\frac{1}{p}\sum_{i=1}^{p}\frac{1}{\left|L\right|}\left|h\left(x_{i}\right)\Delta Y_{i}\right|.$$

This evaluation metric tells how many times an object-label pair is misclassified, where Δ is a binary operator used to calculate the symmetric difference between the $Y_{i}$ and $h\left(x_{i}\right)$. The smaller the indicator value is, the better the performance of the classifier is. The optimal value is 0.

#### 5.2.3. One Error (OE):

(15) $$\mathrm{OE}\left(f\right)=\frac{1}{p}\sum_{i=1}^{p}\left[\left[\arg\max_{y\in Y}f\left(x_{i},y\right)\right]\notin Y_{i}\right].$$

This evaluation metric finds how many times the top-ranked label is not in the set of the proper labels of a sample. The smaller the indicator value is, the better the performance of the classifier is. The optimal value is 0.

#### 5.2.4. Ranking Loss (RL):

(16) $$\mathrm{RL}\left(f\right)=\frac{1}{p}\sum_{i=1}^{p}\frac{1}{\left|Y_{i}\right|\left|Y_{i}^{c}\right|}\left|\left\{\left(y\prime ,y\right)|f\left(\left(x_{i},y\prime \right)\leq \left(x_{i},y^{\prime \prime }\right),\left(y^{\prime },y^{\prime \prime }\right)\in Y_{i}\times Y_{i}^{c}\right)\right\}\right|.$$

This evaluation metric returns the number of times irrelevant labels are ranked higher than relevant ones. The classifier gets the best performance when RL (f)=0.

#### 5.2.5. Coverage (CV):

(17) $$\mathrm{CV}\left(f\right)=\frac{1}{p}\sum_{i=1}^{p}\max_{y\in Y_{i}}rank_{f}\left(x_{i},y\right)-1.$$

This evaluation indicator gives the search depth required to cover all relevant labels in the sample label ordering. The smaller the indicator value is, the better the performance of the classifier is. The optimal value is 0.

### 5.3. Configuration of Related Parameters and Experimental Results

The experiments were run in MATLAB 2012b (MathWorks, Inc., USA). The hardware environment was Intel^®^ Core^TM^i5-3470 3.2-GHz CPU, and the operating system was Windows 7. We compare the proposed SF-DSW-FRL algorithm with feature selection results obtained from seven state-of-the-art algorithms, namely multi-label naive Bayes classification (MLNB) [41], multi-label dimensionality reduction via dependence maximization (MDDMspc) [42], MDDMproj [42], multivariate mutual information criterion for multi-label feature selection (PMU) [18], multi-label feature selection based on neighborhood mutual information (MFNMIpes) [20], MFNMIopt [20], and multi-label feature selection with label correlation (MUCO) [29]. For the MDDMspc algorithm,δ is set to 0.5. In the experiments, *k*NN is used as a classifier, and the ML-*k*NN [43] parameters are set to their default values, that is, the smoothing coefficient is set to 1, and the nearest neighbor number *k* is set to 10. For the proposed SF-DSW-FRL algorithm, the thresholds used with the music, audio, biology, and text datasets are 0.0005, 0.02, 0.06, and 0.01, respectively. The size of the storage window is set to 20. In addition, for all compared algorithms, all features are sorted according to their importance. As our SF-DSW-FRL algorithm directly acquires feature subsets, the same numbers of feature subsets are used for all other algorithms. The feature subset numbers of the eight datasets are 8, 165, 23, 193, 455, 72, 81, and 209, respectively. Table 4, Table 5, Table 6, Table 7 and Table 8 show the predictive performance using the MLNB, MDDMspc, MDDMproj, PMU, MFNMIpes, MFNMIopt, MUCO, and SF-DSW-FRL algorithms, respectively. In the Table, "↑" means *"*the bigger the better" and "↓" means "the smaller the better". The best experimental results are shown in bold. The value in the parenthesis "()" after each experimental result indicates the algorithm ranking on the associated dataset.

The experimental comparison between the SF-DSW-FRL algorithm and the other state-of-the-art algorithms reveal the following:

(1) For the average precision, SF-DSW-FRL achieves the best performance on seven datasets, and the best AP on the Birds dataset is only 0.0176 higher than the AP scored by SF-DSW-FRL. For the Hamming loss, SF-DSW-FRL achieves the best results on six datasets. According to the average ranking results of the eight datasets, SF-DSW-FRL ranks first and performs best.

(2) For the ranking loss, SF-DSW-FRL gets the best score on five data sets. For the other three datasets, the differences between the SF-DSW-FRL scores and the best ones are very small. For example, on the Cal500 dataset, MUCO is the best with an RL value that is only 0.007 lower than that of SF-DSW-FRL. For the one-error metric, SF-DSW-FRL achieves the minimum OE value on three datasets, while its OE scores on the other five datasets are slightly higher than the optimal OE scores.

(3) For the coverage metric whose results are given in Table 8, we realize that SF-DSW-FRL achieves the best minimum coverage score on five datasets.

According to the analysis of the above experimental results in terms of the five evaluation indicators, SF-DSW-FRL is superior to the other algorithms in terms of most evaluation indicators, especially the average precision and the Hamming loss. In addition, SF-DSW-FRL ranks first in the comprehensive ranking of four evaluation indicators. This demonstrates the superiority of the proposed SF-DSW-FRL algorithm. 

### 5.4. Effect and Fine-Tuning of Feature Selection Thresholds

**Figure 2:**
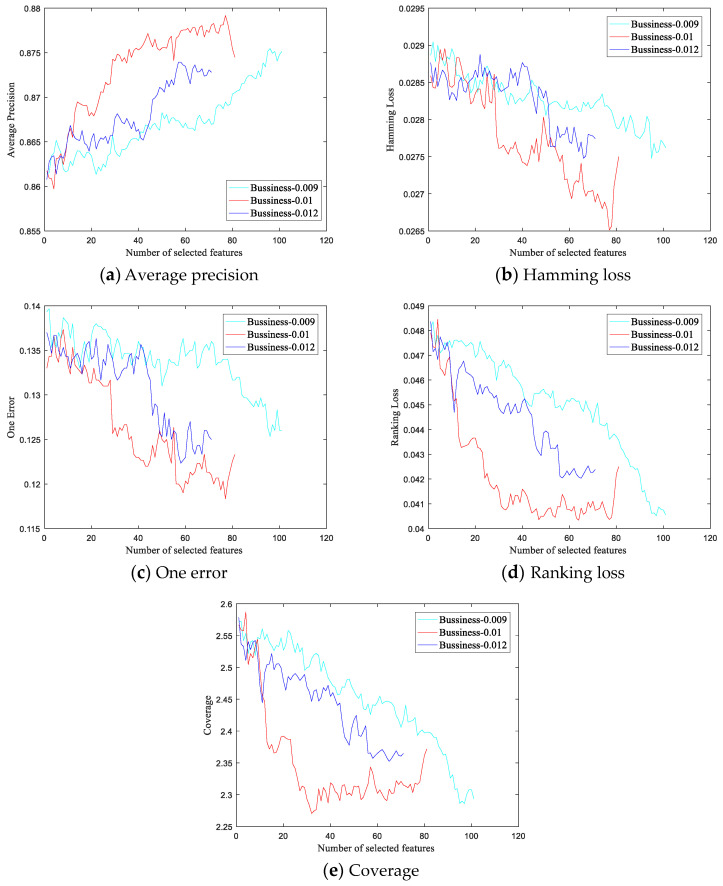
Changes in various evaluation indicators on the Business dataset for different thresholds.

In this section, we evaluate the effect of the feature selection thresholds and fine-tune their values through experiments on the Business dataset. Figure 2a–e shows the classification performance curves of the SF-DSW-FRL method for different thresholds on the five evaluation metrics, respectively. The cyan, red, and blue lines represent the experimental results with thresholds of 0.009, 0.01, and 0.012, respectively. The horizontal and vertical axes represent respectively the number of selected features and the classification performance metrics after feature selection. Among these metrics, the larger the average precision is, the better, while the smaller the other four indicators are, the better.

As shown in Figure 2, variations in threshold values clearly affect the experimental results. The results at a threshold value of 0.009 are the worst. When the threshold value is changed from 0.009 to 0.01, the best results are obtained. The results at a threshold of 0.012 are worse than those at 0.01 but better than those at 0.009. In addition, with the thresholds of 0.009, 0.01, and 0.012, the sizes of the final feature subsets are 101, 81, and 71, respectively. Clearly, the threshold value also affects the size of the final feature subset. The larger the threshold is, the smaller the size of the final feature subset will be.

Based on the above experiments, a threshold of 0.01 was selected for the Business dataset. The experimental results are also shown in Table 4, Table 5, Table 6, Table 7 and Table 8. Threshold selections for seven other datasets are also obtained through similar experiments. The thresholds used with the music (Cal500), audio (Birds), biology (Yeast), and text (Arts, Computer, Health, Business, and Education) datasets are 0.0005, 0.02, 0.06, and 0.01, respectively. The feature subset sizes for the eight datasets are 8, 165, 23, 193, 455, 72, 81, and 209, respectively.

### 5.5. Statistical Hypothesis Testing and Stability Analysis

In order to further verify the effectiveness of the proposed SF-DSW-FRL algorithm, we use the Friedman statistical test [44] with a significance level of 0.05 on the eight datasets. The Friedman test, a non-parametric equivalent of the repeated-measure analysis of variance (ANOVA), ranks the algorithms for each dataset separately. The best performing algorithm receives a rank of 1, the second best receives a rank of 2, and so on (See Table 4, Table 5, Table 6, Table 7 and Table 8). 

**Figure 3:**
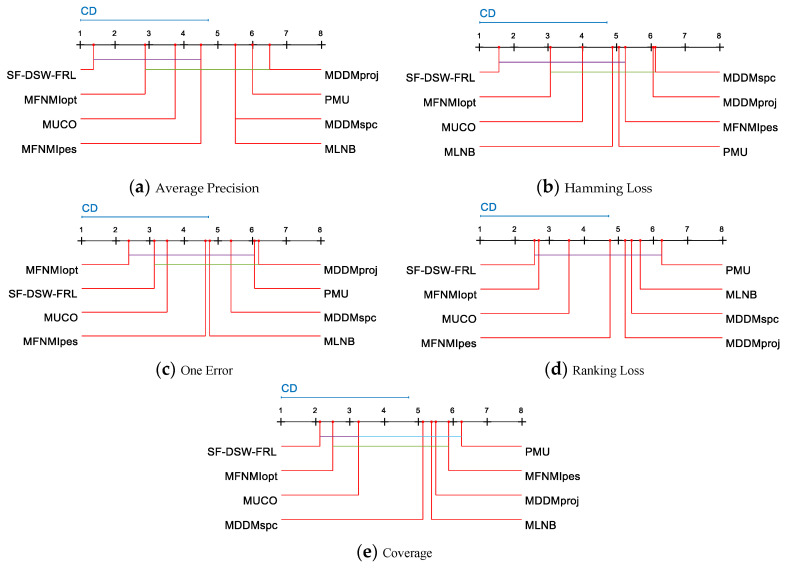
Comparison of the SF-DSW-FRL against other streaming feature selection algorithms with the Friedman statistical test. Critical difference (CD).

When multiple algorithms are compared, the results of the post-hoc tests can be visually represented with a simple diagram [44]. Figure 3a–e visualize the data analysis results for Table 4, Table 5, Table 6, Table 7 and Table 8, respectively.

For each evaluation metric, we reject the null hypothesis and consider the performance of two algorithms to be significantly different if the average ranking results of the two algorithms on all datasets differ by at least a given critical difference (CD) [45]. A single-color line-segment connection indicates no significant performance difference of the two connected algorithms (Figure 3). The average ranking of the algorithms is drawn on the coordinate axis. The smaller the number on the coordinate axis is, the lower the average ranking is. We also show the critical difference (CD) as a blue line above the graph for each performance indicator. The critical difference (CD) is given by

(18) $$CD_{\partial }=q_{\partial }\sqrt{\frac{k\left(k+1\right)}{6N}}.$$

where *k* and *N* are the numbers of algorithms and datasets, respectively, and $q_{\partial }$ is a critical value. According to Equation (18), CD = 3.7122 (*k* = 8, *N* = 8) can be calculated. For each algorithm, there are 35 comparative results (Seven compared algorithms and five evaluation criteria). As shown in Figure 3, we can realize that:

(1) SF-DSW-FRL ranks first in AP, HL, RL, and CV, and it ranks second in OE. (2) For AP and CV, SF-DSW-FRL, MFNMIopt, and MUCO are connected by the same color lines. This means that there is no significant difference among the three algorithms of SF-DSW-FRL, MFNMIopt, and MUCO. SF-DSW-FRL is obviously better than MLNB, MDDMproj, MDDMspc, and PMU in AP and CV. (3) For HL, SF-DSW-FRL has comparable performance with MLNB, MFNMIopt, PMU, MUCO, and MFNMIpes, and it is still significantly better than the other two algorithms. (4) For OE, the performance of SF-DSW-FRL is better than that of MDDMproj. (5) For AP, and HL, the SF-DSW-FRL performance has great advantages over the second-place algorithm. (6) In 34.3% of the cases, SF-DSW-FRL is statistically superior to other compared algorithms. For PMU and MDDMproj, there is no statistical difference between PMU and other algorithms in 88.6% of the cases, while in 11.4% of the cases, PMU is superior to other algorithms. The MDDMspc is statistically superior to other algorithms with a probability of 8.6%, but it shows no significant difference for 91.4% of the cases.

The above analysis shows that SF-DSW-FRL has the best performance. At the level of 34.3%, SF-DSW-FRL is statistically superior to other algorithms. At the level of 65.7%, SF-DSW-FRL has the same performance as other algorithms. The second best methods are PMU and MDDMproj, which are statistically superior to other algorithms at the levels of 11.4%. The above experimental analysis further shows that the performance of SF-DSW-FRL is better in almost all aspects.

The validity and rationality of the proposed algorithm are illustrated by statistical hypothesis testing. The stability of the proposed algorithm is further illustrated by stability analysis.

**Figure 4:**
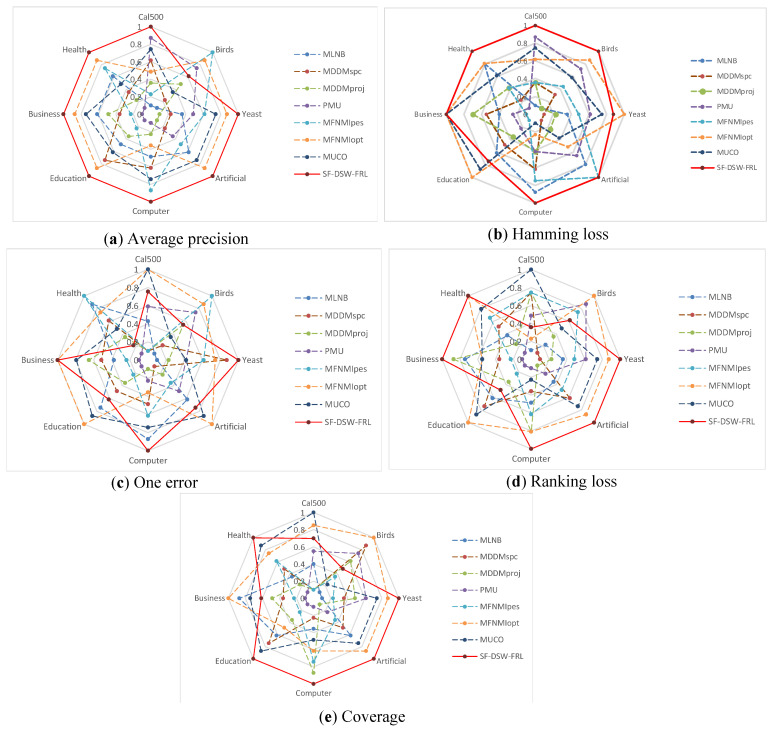
The stability index values obtained on eight benchmark multi-label datasets with different evaluation metrics.

**Stability analysis:** The stability of different multi-label learning algorithms is validated by using spider web graphs. However, the predictive classification performance on different datasets is quite different when no evaluation indicators are used. Therefore, we standardize the prediction classification performance to the range of [0.1,1]. Then, we use the normalized values to represent the stability index. Figure 4a–e shows the stability index of the eight datasets on the five evaluation indicators, respectively.

From Figure 4, we can see: (1) For the average precision, SF-DSW-FRL can obtain the optimal solution on seven datasets. Indeed, the shape of the SF-DSW-FRL polygon is very close to the normal decagon, which means that SF-DSW-FRL obtains a more stable solution. (2) For the Hamming loss, SF-DSW-FRL obtained the optimal solution on six datasets. In addition, for the other two datasets, the stability range was [0.74,0.87]. (3) For the one error metric, the stability index of SF-DSW-FRL on seven datasets is fairly good and is in the range [0.61,1]. (4) For the ranking loss, SF-DSW-FRL achieves a quite stable solution, and it is also more stable than all of the other algorithms. (5) For the coverage metric, SF-DSW-FRL obtained the highest stability on five datasets.

Generally, the results in Figure 4 indicate that SF-DSW-FRL is very stable and has better prediction performance.

## 6. Conclusions

Using a fixed-size sliding window is one effective way to handle streaming data. However, in practical applications, data is generated in real time, and the number of generated data samples varies with time. As well, the algorithms proposed to handle multi-label streaming feature selection seldom consider the group structure of streaming features. To remedy these shortcomings, we develop the streaming feature selection algorithm with dynamic sliding windows and feature repulsion loss (SF-DSW-FRL). In particular, the streaming feature selection problem is handled through a combination of fixed and dynamic sliding windows. For the dynamic sliding window, group flow features are processed to account for the interaction between features, select the features that have a strong correlation with the data labels, and store these features in a fixed sliding window. In the fixed sliding window, the importance of candidate features is measured based on the feature repulsion loss. The experimental results and the statistical hypothesis testing results illustrate the effectiveness of the proposed algorithm. One disadvantage of the proposed method is the manual setting of the threshold of the dynamic sliding window. In future work, parameters will be fine-tuned to improve the feature selection performance.

## Figures and Tables

**Table 1:** Toy example of a multi-label task.

*U*	$y_{i}$
$x_{1}$	$l_{1}$,$l_{3}$
$x_{2}$	$l_{2}$,$l_{3}$
$x_{3}$	$l_{1}$
$x_{4}$	$l_{1}$,$l_{2}$

**Table 2:** Binary representation of a multi-label task.

*U*	$l_{1}$	$l_{2}$	$l_{3}$
$x_{1}$	1	0	1
$x_{2}$	0	1	1
$x_{3}$	1	0	0
$x_{4}$	1	1	0

**Table 3:** Experimental datasets for the streaming feature selection algorithm with dynamic sliding windows and feature repulsion loss (SF-DSW-FRL).

Datasets	Objects	Features	Classes	Training	Test	Domain
Cal500	502	68	174	251	251	Music
Birds	645	260	20	322	323	Audio
Yeast	2417	103	14	1499	918	Biology
Artificial	5000	462	26	2000	3000	Text
Computer	5000	681	33	2000	3000	Text
Health	5000	612	32	2000	3000	Text
Business	5000	438	30	2000	3000	Text
Education	5000	550	33	2000	3000	Text

**Table 4:** Precision performance for eight feature selection methods (↑).

Methods	MLNB	MDDMspc	MDDMproj	PMU	MFNMIpes	MFNMIopt	MUCO	SF-DSW-FRL
Cal500	0.4603(8)	0.4730(4)	0.4720(6)	0.4753(2)	0.4702(7)	0.4723(5)	0.4739(3)	**0.4812**(1)
Birds	0.6756(8)	0.6822(7)	0.6904(5)	0.7009(3)	**0.7082**(1)	0.7070(2)	0.6902(6)	0.6906(4)
Yeast	0.7219(6)	0.7204(7)	0.7187(8)	0.7282(5)	0.7286(4)	0.7444(2)	0.7325(3)	**0.7458**(1)
Artificial	0.5118(4)	0.4956(7)	0.4907(8)	0.5036(6)	0.5041(5)	0.5217(2)	0.5168(3)	**0.5221**(1)
Computer	0.6302(5)	0.6311(4)	0.6284(7)	0.6264(8)	0.6335(2)	0.6295(6)	0.6312(3)	**0.6368**(1)
Education	0.5289(5)	0.5363(3)	0.5107(6)	0.4898(8)	0.4937(7)	0.5386(2)	0.5362(4)	**0.5405**(1)
Business	0.8711(4)	0.8685(6)	0.8700(5)	0.8605(8)	0.8634(7)	0.8743(2)	0.8739(3)	**0.8745**(1)
Health	0.6880(4)	0.6821(6)	0.6708(7)	0.6560(8)	0.6915(3)	0.6950(2)	0.6828(5)	**0.6986**(1)
***Average***	5.5(5.5)	5.5(5.5)	6.5(8)	6(7)	4.5(4)	2.875(2)	3.75(3)	**1.375**(1)

**Table 5:** Hamming loss performance for eight feature selection methods (↓).

Methods	MLNB	MDDMspc	MDDMproj	PMU	MFNMIpes	MFNMIopt	MUCO	SF-DSW-FRL
Cal500	0.1432(8)	0.1417(6)	0.1417(6)	0.1402(2)	0.1417(6)	0.1407(4)	0.1405(3)	**0.1394**(1)
Birds	0.0565(7.5)	0.0556(6)	0.0565(7.5)	0.0533(3)	0.0545(5)	0.0531(2)	0.0534(4)	**0.0526**(1)
Yeast	0.2227(6)	0.2263(8)	0.2228(7)	0.2158(4)	0.2172(5)	**0.2078**(1)	0.2130(3)	0.2079(2)
Artificial	0.0599(3)	0.0613(8)	0.0609(7)	0.0601(4)	**0.0598**(1.5)	0.0602(5)	0.0605(6)	**0.0598**(1.5)
Computer	0.0403(2)	0.0409(4)	0.0410(5.5)	0.0410(5.5)	0.0408(3)	0.0411(7)	0.0412(8)	**0.0402**(1)
Education	0.0424(4)	0.0431(5)	0.0436(6)	0.0442(7)	0.0443(8)	**0.0407**(1)	0.0420(2)	0.0421(3)
Business	0.0283(6)	0.0282(5)	0.0281(4)	0.0286(7)	0.0287(8)	**0.0275**(2)	**0.0275**(2)	**0.0275**(2)
Health	0.0429(2.5)	0.0435(7)	0.0434(5.5)	0.0468(8)	0.0434(5.5)	0.0429(2.5)	0.0432(4)	**0.0427**(1)
***Average***	4.875(4)	6.125(8)	6.0625(7)	5.0625(5)	5.25(6)	3.0625(2)	4(3)	**1.5625**(1)

**Table 6:** One error performance for eight feature selection methods (↓).

Methods	MLNB	MDDMspc	MDDMproj	PMU	MFNMIpes	MFNMIopt	MUCO	SF-DSW-FRL
Cal500	0.1235(5)	0.1514(7)	0.1514(7)	0.1176(4)	0.1514(7)	**0.1116**(1.5)	**0.1116**(1.5)	0.1155(3)
Birds	0.4025(8)	0.3941(7)	0.3932(4.5)	0.3777(3)	**0.3406**(1)	0.3498(2)	0.3939(6)	0.3932(4.5)
Yeast	0.2658(8)	0.2473(2)	0.2603(7)	0.2593(5.5)	0.2505(4)	0.2495(3)	0.2593(5.5)	**0.2429**(1)
Artificial	0.6250(4)	0.6700(8)	0.6480(7)	0.6343(5)	0.6353(6)	**0.5947**(1)	0.6063(2)	0.6130(3)
Computer	0.4347(2)	0.4427(5)	0.4497(8)	0.4493(7)	0.4417(4)	0.4450(6)	0.4415(3)	**0.4320**(1)
Education	0.6007(3)	0.6123(5)	0.6380(6)	0.6720(8)	0.6703(7)	**0.5513**(1)	0.5887(2)	0.6110(4)
Business	0.1318(6)	0.1310(5)	0.1303(4)	0.1367(8)	0.1340(7)	**0.1233**(1.5)	0.1240(3)	**0.1233**(1.5)
Health	0.3947(2)	0.4073(4)	0.4213(6)	0.4397(8)	**0.3873**(1)	0.3973(3)	0.4083(5)	0.4240(7)
***Average***	4.75(5)	5.375(6)	6.1875(8)	6.0625(7)	4.625(4)	**2.375**(1)	3.5(3)	3.125(2)

**Table 7:** Ranking loss performance for eight feature selection methods (↓).

Methods	MLNB	MDDMspc	MDDMproj	PMU	MFNMIpes	MFNMIopt	MUCO	SF-DSW-FRL
Cal500	0.1978(8)	0.1903(3)	0.1903(3)	0.1905(5)	0.1903(3)	0.1933(7)	**0.1899**(1)	0.1906(6)
Birds	0.1422(7)	0.1432(8)	0.1362(6)	0.1214(2)	0.1297(3)	**0.1205**(1)	0.1345(5)	0.1309(4)
Yeast	0.1971(6)	0.2022(8)	0.2013(7)	0.1924(4)	0.1957(5)	0.1833(2)	0.1913(3)	**0.1826**(1)
Artificial	0.1537(6)	0.1530(4)	0.1554(8)	0.1553(7)	0.1531(5)	0.1460(2)	0.1467(3)	**0.1456**(1)
Computer	0.0925(5)	0.0927(6)	0.0918(2.5)	0.0944(8)	0.0920(4)	0.0918(2.5)	0.0931(7)	**0.0916**(1)
Education	0.0939(4)	0.0933(3)	0.1008(6)	0.1058(8)	0.1036(7)	**0.0903**(1)	0.0931(2)	0.0952(5)
Business	0.0429(3)	0.0445(6)	0.0428(2)	0.0487(8)	0.0460(7)	0.0430(4.5)	0.0430(4.5)	**0.0425**(1)
Health	0.0641(6)	0.0624(5)	0.0653(7)	0.0715(8)	0.0614(4)	**0.0598**(1.5)	0.0613(3)	**0.0598**(1.5)
***Average***	5.625(7)	5.375(6)	5.1875(5)	6.25(8)	4.75(4)	2.6875(2)	3.5625(3)	**2.5625**(1)

**Table 8:** Coverage performance for eight feature selection methods (↓).

Methods	MLNB	MDDMspc	MDDMproj	PMU	MFNMIpes	MFNMIopt	MUCO	SF-DSW-FRL
Cal500	132.3944(5)	132.4741(7)	132.4741(7)	132.0400(4)	132.4741(7)	130.8008(2)	**130.4263**(1)	131.4622(3)
Birds	3.6842(8)	3.2724(2)	3.4025(4)	3.2817(3)	3.5697(6)	**3.2539**(1)	3.6533(7)	3.4954(5)
Yeast	6.9455(8)	6.8126(6)	6.7930(5)	6.7516(4)	6.8366(7)	6.6166(2)	6.6481(3)	**6.5044**(1)
Artificial	5.3313(4)	5.4837(5)	5.5620(8)	5.5533(7)	5.5273(6)	5.3073(2)	5.3157(3)	**5.2807**(1)
Computer	4.4487(6)	4.4713(7)	4.4053(2)	4.5313(8)	4.4187(3)	4.4303(4)	4.4358(5)	**4.4080**(1)
Education	3.9670(4)	3.9610(3)	4.1927(6)	4.3967(8)	4.3237(7)	3.9787(5)	3.9533(2)	**3.8900**(1)
Business	2.3483(2)	2.4383(6)	2.3927(5)	2.5837(8)	2.5123(7)	**2.3000**(1)	2.3567(3)	2.3717(4)
Health	3.4163(6)	3.3737(5)	3.4713(7)	3.7030(8)	3.3523(4)	3.3163(3)	3.3103(2)	**3.3033**(1)
***Average***	5.375(5)	5.125(4)	5.5(6)	6.25(8)	5.875(7)	2.5(2)	3.25(3)	**2.125**(1)
